# *In vitro* susceptibility of *Cutibacterium acnes* to dalbavancin: comparison between gradient test and the agar dilution reference method

**DOI:** 10.1093/jac/dkag364

**Published:** 2026-10-22

**Authors:** Sara Svensson, Susanne Jacobsson, Erika Matuschek, Bo Söderquist

**Affiliations:** School of Medical Sciences, Faculty of Medicine and Health, Örebro University, Örebro, Sweden; Department of Orthopedics, Faculty of Medicine and Health, Örebro University, Örebro, Sweden; Department of Laboratory Medicine, WHO Collaborating Centre for Gonorrhoea and Other STIs, National Reference Laboratory for STIs, Faculty of Medicine and Health, Örebro University, Örebro, Sweden; EUCAST Development Laboratory, Clinical Microbiology, Växjö Hospital, Växjö, Sweden; School of Medical Sciences, Faculty of Medicine and Health, Örebro University, Örebro, Sweden; Department of Orthopedics, Faculty of Medicine and Health, Örebro University, Örebro, Sweden

## Abstract

**Background:**

*Cutibacterium acnes*, a human commensal, occasionally causes infections requiring long-term antibiotic treatment, including periprosthetic joint infections. Dalbavancin is a new lipoglycopeptide that allows intermittent dosing because of its exceptionally long half-life. However, data on susceptibility of *C. acnes* to dalbavancin are lacking, and no EUCAST breakpoints have been defined.

**Objectives:**

To determine the MIC distribution of *C. acnes* for dalbavancin, and to compare the gradient test to the agar dilution reference method.

**Methods:**

*C. acnes* isolates were obtained from tissue samples from arthroplasties (*n* = 117), skin samples (*n* = 15), and positive blood cultures (*n* = 85). Antibiotic susceptibility was tested by agar dilution according to EUCAST methodology for anaerobic bacteria (±polysorbate 80) and by gradient test.

**Results:**

MIC_50_ and MIC_90_ for dalbavancin against *C. acnes* were 0.03 and 0.06 mg/L using agar dilution, and 0.004 and 0.008 mg/L with the gradient test. The gradient test yielded 3-fold lower MIC values than agar dilution. No significant difference was observed when polysorbate 80 was added to the agar dilution test. When vancomycin was tested as a comparator, the MICs did not differ between the agar dilution and gradient tests; MIC_50_ and MIC_90_ were 0.5 mg/L in both tests.

**Conclusions:**

*C. acnes* showed significantly lower MIC values for dalbavancin than for vancomycin, highlighting the potential of dalbavancin as a therapeutic option. The gradient test yielded significantly lower MICs for dalbavancin than the reference method for anaerobes, agar dilution, suggesting that the gradient test for dalbavancin may not be a reliable method for antibiotic susceptibility testing (AST) of *C. acnes*.

## Introduction

*Cutibacterium acnes*, a human commensal, occasionally causes infections requiring long-term antibiotic treatment, including infective endocarditis and periprosthetic joint infections (PJIs).^1,2^

Dalbavancin is a semisynthetic lipoglycopeptide approved by the EMA for use in acute bacterial skin and skin-structure infections.^3^ Its large lipid chain gives it an exceptionally long half-life of 204 h and a terminal elimination half-life of 346 h,^4^ which allows intermittent administration. However, its documented effect on Gram-positive bacteria^5^ has led to an increasing use of off-label treatment with dalbavancin for serious infections that require long-term therapy, including orthopaedic infections such as PJIs.^6^

EUCAST defines, harmonizes, and standardizes antimicrobial susceptibility breakpoints and testing methods. According to EUCAST, the breakpoints for vancomycin against *C. acnes* are susceptible ≤2 mg/L and resistant >2 mg/L, but data on the susceptibility of *C. acnes* to dalbavancin are lacking, and no breakpoints have yet been defined.^7^

The gold standard method for susceptibility testing of anaerobes is agar dilution.^8^ However, MIC determination with agar dilution for dalbavancin has not been validated. In addition, the gradient test method is a more accessible and efficient option for routine laboratory testing.^9^ A recent study concluded that gradient diffusion is an acceptable alternative to broth microdilution for susceptibility testing of aerobic cocci, such as staphylococci and enterococci, against dalbavancin, daptomycin, vancomycin, and teicoplanin.^10^ Another study found that for anaerobic bacteria, the gradient test can generate lower MIC values than the agar dilution method.^11^ However, to our knowledge, no data are available on the correlation between agar dilution and gradient tests for *C. acnes* and dalbavancin.

The aim of this study was to develop MIC distributions for *C. acnes* and dalbavancin, and to examine whether the gradient test is comparable to the agar dilution reference method.

## Material and methods

### Bacterial isolates

This study included 217 anonymized *C. acnes* isolates collected from positive tissue samples from arthroplasties (*n* = 117), skin samples (*n* = 15), and positive blood cultures (*n* = 85). Isolates were stored in pure culture at −80°C in a preservation medium composed of trypticase soy broth enriched with 0.3% yeast extract and 29% horse. All isolates were stored in pure culture at the Department of Laboratory Medicine, Clinical Microbiology, Örebro University Hospital, Sweden, according to local routine procedures.

### Preparation of isolates

*C. acnes* samples were anaerobically cultured for 3 days at 36°C on fastidious anaerobe agar with 5% horse blood (FAA-HB) plates (4.6% [w/v] LAB 90 Fastidious Anaerobe Agar, Neogen, Heywood, Lancashire, UK, supplemented with 5% defibrinated horse blood, Håtunalab AB). Species identification was performed using MALDI-TOF MS (Microflex LT and Biotyper 3.1, Bruker Daltonik, Bremen, Germany).

### Agar dilution method

The MICs (mg/L) of dalbavancin and vancomycin were determined by agar dilution according to the CLSI guidelines^12^ using FAA-HB, which is the EUCAST-recommended medium.^13^ Three sets of FAA-HB plates were prepared: the first set supplemented with dalbavancin at concentrations ranging from 0.004 to 4 mg/L, the second set with dalbavancin at concentrations ranging from 0.004 to 4 mg/L plus 0.002% polysorbate (Tween-80, Merck, Darmstadt, Germany), and the third set with vancomycin at concentrations ranging from 0.004 to 4 mg/L. Antibiotic-free plates served as growth control.

Bacterial suspensions were adjusted to 0.5 McFarland in sterile 0.85% NaCl and inoculated (10^5^ CFU per spot, up to 36 bacterial isolates per plate) using a Multipointelite inoculator (Mast Group Ltd, Bootle, UK). For quality control, the reference strains *Staphylococcus aureus* ATCC 29213 and *Clostridium perfringens* ATCC 13124 were included in each run. Plates were incubated in an anaerobic atmosphere (80% N_2_, 10% CO_2_, 10% H_2_) at 36°C for 42–48 h.

### Gradient test

The MICs (mg/L) of dalbavancin and vancomycin were also determined using MIC gradient tests according to the manufacturer’s instructions. Briefly, a bacterial suspension of 1.0 McFarland was prepared in sterile 0.85% NaCl and distributed onto FAA-HB plates. Dalbavancin MIC test strips (MTS) (Liofilchem, Roseto degli Abruzzi, Italy) and vancomycin Etest strips (bioMérieux, Marcy l’Etoile, France) were applied, and the plates were incubated anaerobically at 36°C and read after 24 and 48 h.

### Statistics

The Wilcoxon signed-rank test was used to compare the paired methods (IBM SPSS Statistics, version 30, IBM, Corp., Armonk, NY, USA). Statistical significance was set at *P* < 0.05. Essential agreement (MICs being within ± 1 dilution) and bias (whether results deviating from the reference tests are skewed versus higher or lower MICs) were calculated according to ISO 20776-2:2021 using agar dilution as reference. The acceptance criteria were essential agreement ≥90% and bias <±30%.

## Results

The gold-standard agar dilution method showed that dalbavancin against *C. acnes* had a MIC range of 0.016–0.125 mg/L and MIC_50_ and MIC_90_ values of 0.03 and 0.06 mg/L, respectively. However, the gradient test showed a MIC range of <0.002–0.008 mg/L and MIC_50_ and MIC_90_ values of 0.004 mg/L and 0.008 mg/L, respectively (Figure 1). Thus, there was a significant difference between the gradient test and agar dilution method, with the gradient test yielding MIC values that were 3-fold lower than those obtained using the agar dilution method (*P* < 0.001). Addition of polysorbate 80 to the agar dilution plates did not affect the MICs (Figures S1 and S2, available as Supplementary data at *JAC* Online)

**Figure 1.**
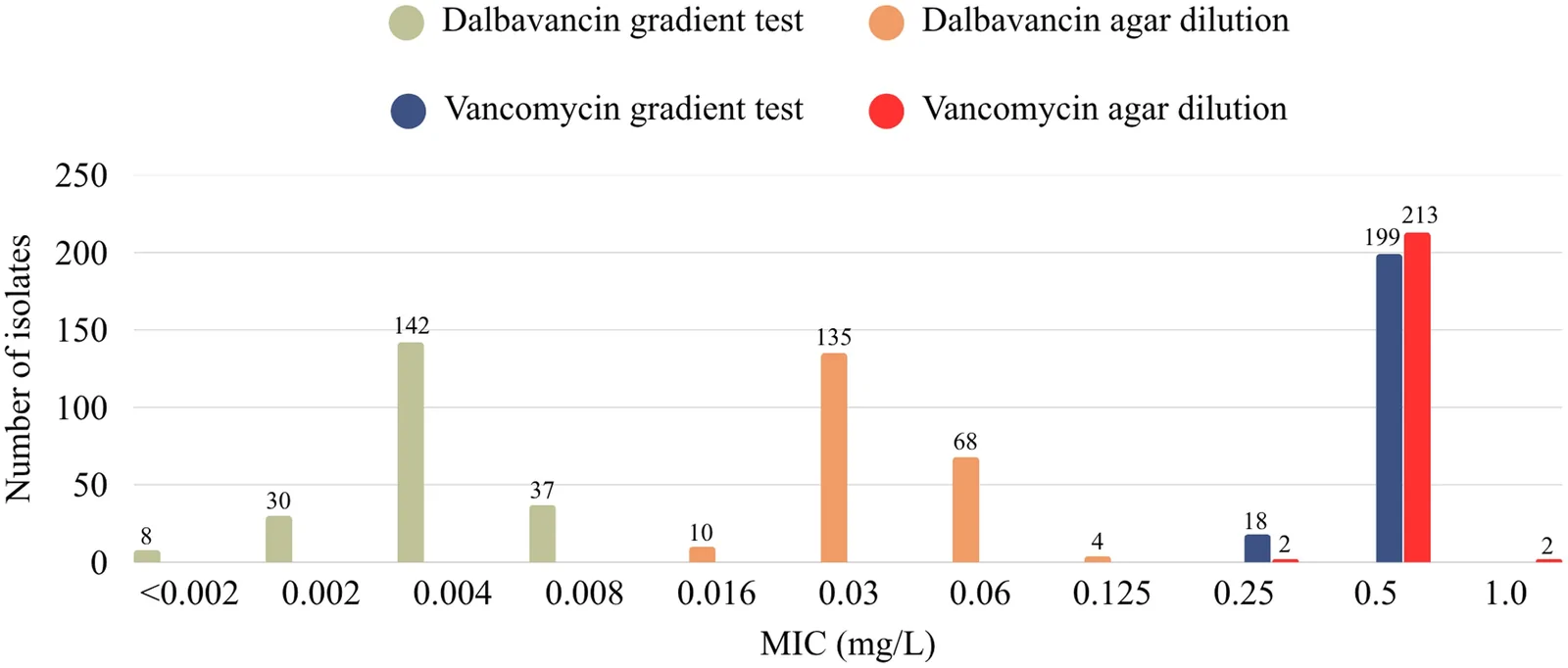
MIC distribution of *C. acnes* determined by gradient test and agar dilution, tested with dalbavancin and vancomycin.

For vancomycin, the MIC range was 0.25–1 mg/L when the agar dilution method was used and 0.25–0.5 mg/L when the gradient test method was used. The MIC_50_ and MIC_90_ values were both 0.5 mg/L, regardless of whether agar dilution or gradient test was used. Dalbavancin had a >3-fold lower MIC than vancomycin for both agar dilution (*P* < 0.001) and the gradient test (*P* < 0.001) (Figure 1).

The essential agreement for the vancomycin Etest was 100%, with a low bias (−8.3%), rendering all results within the acceptance criteria of ISO 20776-2:2021 (Figure 2).

**Figure 2.**
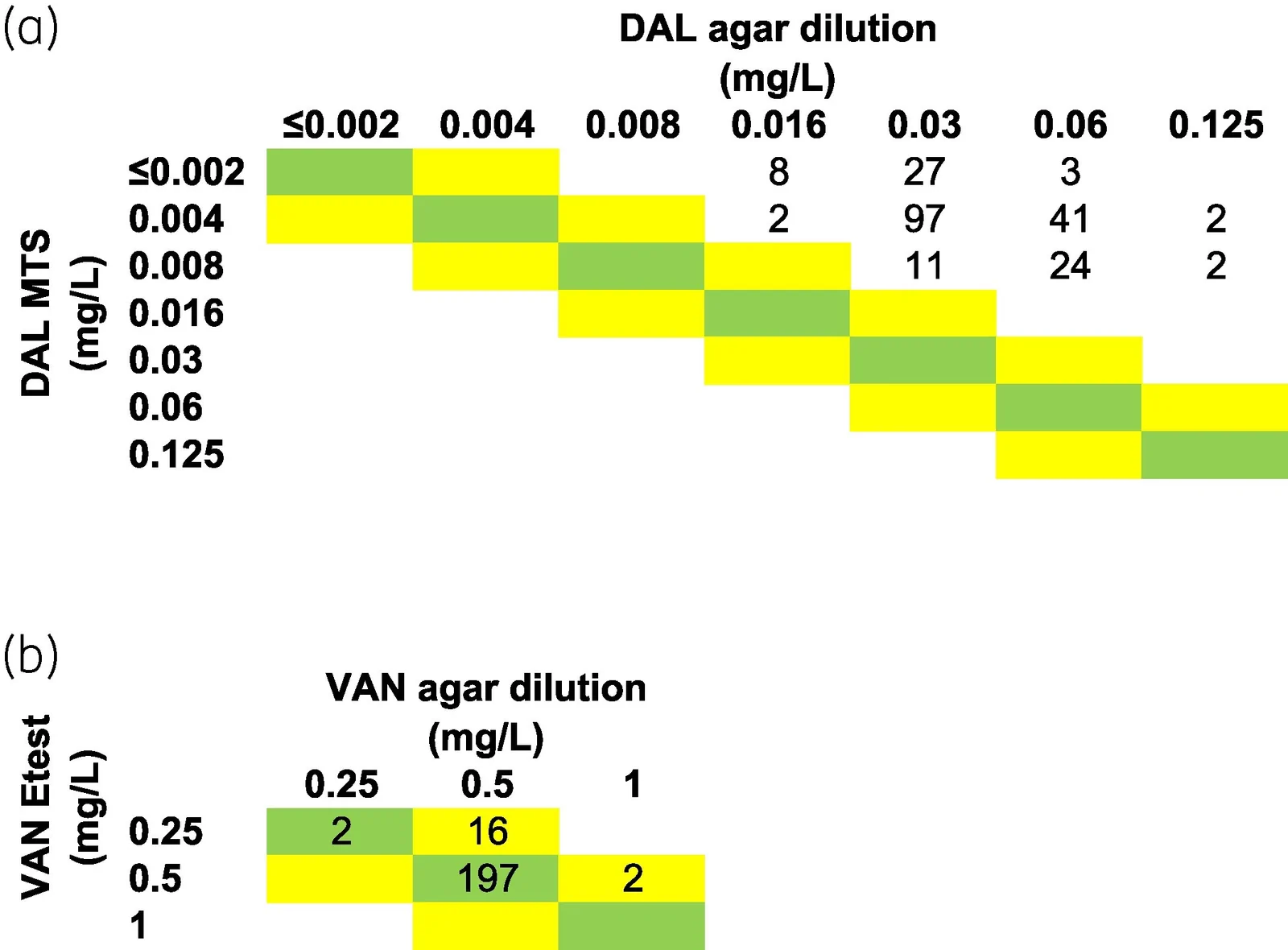
Essential agreement and bias for (a) dalbavancin determined by gradient test (MTS) versus agar dilution and (b) for vancomycin tested using the gradient test (Etest) versus agar dilution method. DAL,  dalbavancin; MTS, MIC test strip. Green boxes show identical MICs and yellow boxes show MICs within one doubling dilution.

A comparison of the isolates by site of origin is shown in Figure S3.

## Discussion

The present study primarily aimed to determine the MIC distribution of *C. acnes* for dalbavancin and, for comparison, to assess susceptibility to vancomycin. In addition, we aimed to evaluate the routinely used gradient test in comparison with the gold standard agar dilution method for susceptibility testing for anaerobic bacteria.

In this study, all *C. acnes* isolates tested for dalbavancin exhibited MIC values below 0.125 mg/L, with 90% of the isolates having MICs below 0.06 mg/L. These findings are consistent with those of a previous study that reported a higher susceptibility of Gram-positive anaerobes to dalbavancin than to other antibiotics, where 91% of the tested isolates had MICs <1 mg/L.^14^ This comparison supports the suggestion that *C. acnes* is likely to be susceptible to dalbavancin,^13^ as our results showed even lower MIC values than those found for other anaerobic bacteria. However, the MIC is a relative value, irrespective of the method used, and the activity of an antimicrobial agent cannot be determined solely by MIC values. Instead, activity is determined by applying PK/PD data derived from relevant *in vitro* and *in vivo* infection models.^15^

To our knowledge, this is the first published comparison of the agar dilution method and the gradient test for *C. acnes* and dalbavancin. However, a previous study found that the gradient test and agar dilution method were comparable for susceptibility testing of anaerobic bacteria against several antibiotic classes, showing agreement within 1–2 dilution steps.^16^ Discrepancies between the two methods have also been described, indicating that the agar dilution method generally yielded higher MICs for all tested isolates.^9^ This aligns with the results of the present study, where a 3-fold difference in dilution steps was observed for dalbavancin. However, as far as we know, the dalbavancin gradient test (MTS) has not been validated by the manufacturer for MIC determination in anaerobic bacteria.^17^ By contrast, no differences were observed between the gradient test and agar dilution for vancomycin. Since previous studies have shown inconsistent agreement between the methods, this variation may reflect differences depending on the evaluated antibiotic.

*C. acnes* yielded lower MIC values for dalbavancin than for vancomycin, as determined by agar dilution. All isolates were below the defined vancomycin breakpoint (S ≤ 2 mg/L). Dalbavancin has been reported to be 1–3 dilution steps more active than vancomycin.^15^ However, our results showed even greater differences; 2–5 dilution steps lower with the agar dilution method compared to vancomycin, suggesting a high potency of dalbavancin against *C. acnes*.^15^ Since all isolates were susceptible to vancomycin,^7^ these findings imply that the isolates may also be susceptible to dalbavancin, and all isolates seem to belong to the dalbavancin wild-type population. However, official breakpoints for *C. acnes* have not yet been established. The present data may provide a potential basis for establishing an epidemiological cut-off value as well as clinical breakpoints for dalbavancin and *C. acnes*.

### Conclusions

*C. acnes* showed significantly lower MIC values for dalbavancin than for vancomycin, highlighting the potential of dalbavancin as a therapeutic option. The gradient test yielded significantly lower MICs for dalbavancin than the reference method for anaerobes, agar dilution, suggesting that the gradient test for dalbavancin may not be a reliable method for AST of *C. acnes*.

## Supplementary Material

dkag364_Supplementary_Data

## Supplementary data

Figures S1, S2 and S3 are available as Supplementary data at JAC Online.
