# Supplementary material for: In vitro susceptibility of Cutibacterium acnes to dalbavancin: comparison between gradient test and the agar dilution reference method
Source: J Antimicrob Chemother. 2026 Oct 22;81(11):dkag364. doi: 10.1093/jac/dkag364 (PMC13687895; doi:10.1093/jac/dkag364)
Supplement: Supplementary file 1 [file dkag364_supplementary_data.docx]

Supplementary Material

**Appendix 1.** Comparing dalbavancin agar dilution MICs with and without polysorbate 80, the results of the agar dilution method were not altered by the addition of polysorbate 80 to the agar (the Wilcoxon signed-rank test; p=0.202) (Figure S1), and the essential agreement was 99.5% and the bias was -1.4% (Figure S2), confirming that there was no systematic difference in dalbavancin MICs determined with and without polysorbate 80. Agar dilution without polysorbate 80 was therefore used as reference when evaluating the gradient test.

**Figure S1.** MIC distribution of dalbavancin for C. acnes (n=217) determined by gradient test and agar dilution method with and without 0.002% polysorbate 80.

***Figure S2.*** *Essential agreement and bias for dalbavancin for agar dilution with polysorbate 80 vs. agar dilution without polysorbate 80. DAL = dalbavancin. Green boxes show identical MICs and yellow boxes show MICs within one doubling dilution.*

**Appendix 2.** Comparing the isolates by site of origin, a significant difference in the MIC distribution of dalbavancin determined by the agar dilution method was observed between the joint (n=117) and blood isolates (n=85) (the Mann–Whitney U test; p<0.001), but not between the joint and skin isolates (n=15) or between the skin and blood isolates (Figure 5). Within the joint isolates (knee, n=18; hip, n=33; shoulder, n=65; and ankle, n=1), no statistically significant differences were observed between knee, hip, and shoulder sites of origin (ankle isolate excluded due to insufficient number of isolates).

**Figure S3.** MIC distribution of dalbavancin determined by the agar dilution method for C. acnes isolated from tissue samples of prosthetic joints, blood cultures, and skin of healthy individuals.
